# Potential biomarkers in *Japanese encephalitis* from different hosts and geographical locations

**DOI:** 10.6026/97320630019611

**Published:** 2023-05-31

**Authors:** Sain Ziaullah M, Mohammad Azhar Kamal, Mohiuddin Khan Warsi, Saad Alghamdi, Mohammed Yahya Al Qahtani, Ahmed Muhammed Al Rumaihi, Asif Hussain Akber, Mohammed Ali Al Qahtani, Misbahuddin M Rafeeq

**Affiliations:** 1 Department of Microbiology, Faculty of Medicine, Rabigh, King Abdulaziz University, Jeddah KSA 21589; 2 Department of Pharmaceutics, College of Pharmacy, Prince Sattam Bin Abdulaziz University, Alkharj 11942, Saudi Arabia; 3 Department of Biochemistry, College of Science, University of Jeddah, Jeddah 23890, Saudi Arabia; 4 University of Jeddah Centre for Scientific and Medical Research (UJ-CSMR), University of Jeddah, Jeddah, Saudi Arabia; 5 Laboratory Medicine Department, Faculty of Applied Medical Sciences, Umm Al-Qura University, Makkah, Saudi Arabia; 6 Central Military Laboratory and Blood Bank Department - Virology Division, Prince Sultan Military Medical City, Riyadh 12233, Saudi Arabia; 7 Central Military Laboratory and Blood Bank Department - Virology Division, Prince Sultan Military Medical City, Riyadh 12233, Saudi Arabia; 8 Central Military Laboratory and Blood Bank Department - Virology Division, Prince Sultan Military Medical City, Riyadh 12233, Saudi Arabia; 9 Central Military Laboratory and Blood Bank Department - Microbiology Division, Prince Sultan Military Medical City, Riyadh 12233, Saudi Arabia; 10 Department of Pharmacology, Faculty of Medicine, Rabigh, King Abdulaziz University Jeddah, 21589, KSA

**Keywords:** *Japanese encephalitis* virus, Single Nucleotide Polymorphism (SNPs), transcription factors, phobos, phylogenomics

## Abstract

*Japanese encephalitis* (JE) is a single-stranded, mosquito-borne, positive-sense RNA flavivirus that causes one of the most severe encephalitides. There are treatments available for those who contact this illness; however, there are no known cures. This disease has a 30% fatality rate, and of the people who survive, 30-50% develops neurologic and psychiatric sequelae. The JE virus genome size is 10.98 kb and contains two coding DNA sequences (CDS), two genes, and 15 mature peptides; the CDS polyprotein is 10.3 kb. In this study, we used 29 genomics sequences of the JE virus reported from different countries and infecting different animals and analysed vast dimensions of the genomic annotation of JE comparatively to understand its evolutionary aspects. The extensive SNPs analysis revealed that KF907505.1, reported from Taiwan, has only three SNPs, similar to sequences reported from India. Repeat and polymorphism analyses revealed that the genome tends to be similar in most JE sequences.

## Background:

The *Japanese encephalitis* (JE) virus is a vector-borne virus belonging to the Flaviviridae family. It causes a fatal form of encephalitis that affects people in Asia, Western Pacific nations, and northern Australia [1, 2]. In 29 JE epidemic countries, 67,900 JE cases have been estimated annually, and in 2011 alone, a total of 10,426 cases were reported by governments and other agencies. JE is not a new threat to humans, as it is an old virus that causes infection in many animals. A few examples include Armigeres subalbatus, Bos taurus, Culex pseudovishnui, Culex tritaeniorhynchus, Equus caballus, pig, Sus scrofa, and other species [3-4. 5]. Although the first case of JE virus disease was reported in 1871 in Japan, it is presently found in oversized proportions in most Asian countries, including India. Pigs and different wild birds are the infection's characteristic repository because its enzootic cycle is present among mosquitoes and vertebrate hosts. Pigs and other animals transmit it to humans through infected mosquitoes from the Culex species, especially Cu-lex tritaeniorhynchus [6-7]. Genome sequencing is an essential tool in research that helps us find information about an organism. While scientists from many countries have reported genome sequences and submitted them to the NCBI, many are still being sequenced and analysed worldwide. Most published research suggests that only a few drugs work effectively against JE; however, there is still no drug to cure this disease [8-9,10]. There are tremendous complications involved in designing a drug against any virus due to its mutational adaptation and modifications in its genomic islands. In this study, we aimed to understand the diversity of the genome in JE viruses and to study how divergent it is based on its infectiousness or the infecting organisms. We have extensively collected data, including reporting country, reporting year, and host organism, to mine various constraints of the genomic sequences and chose the broadly different data of JE from Japan, China, Taiwan, and India to find the exact mutational adaptation. We have included data from different hosts to understand the genomic divergence between all species that infect other hosts. Further, we have created a local database for the genomic re-annotation within all species, resulting in various new annotations from the genome, and extracted the data to plot them in a graphical format to understand its multiple restraints. We have also identified the restriction sites on the genomes and categorised the sequence repeats into long sequence repeats (LSRs) and the short sequence repeats (SSRs) that can be used as biomarkers for drug targets and molecular characterisation.

## Global Reporting on *Japanese encephalitis*:

**Figure 1:**
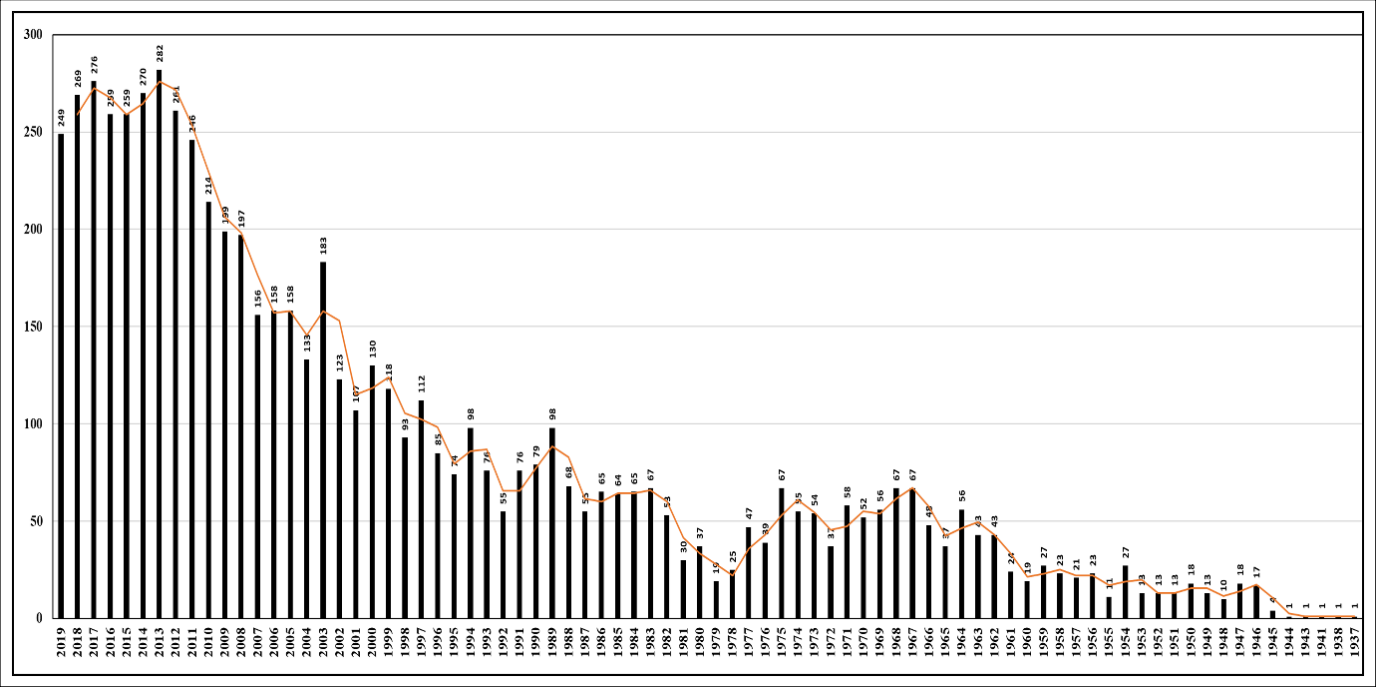
Global reporting of *Japanese encephalitis*, year-wise reporting from worldwide in PubMed. The highest number of publications was reported in 2013.

JE infection is the principal source of viral encephalitis in Asian countries. It occurs chiefly in provincial rural areas where the flooding irrigation system is not advanced, some of which might be close to or inside urban centres [11]. Most transmissions spread during the wet season in Southeast Asia. However, it may occur throughout the year, especially in tropical atmospheric zones. In the temperate areas of China, Japan, the Korean peninsula, and the eastern regions of the Russian Federation, it is transmitted predominantly throughout summer and autumn [11-12, 13]. Because of immunisation, infection rates have decreased in Japan, some regions of China, and Korea. The transmission of the infection is not affected by immunisation, and non-immunised people are still at risk of infection. The disease has also been reported globally in Bangladesh, Pakistan, Cambodia, the Philippines, and other nations. In recent years, the infection rate of JE has increased in India [11]. Because of its colossal spread and high death rates, scientists are working worldwide to find a cure and publish the reports; most of them have been listed in PubMed, which has been used to analyse global reporting. While exploring the PubMed publications, we found that 6,766 reports were submitted from 1937 to 2019, which shows exponential growth in research related to this virus. Before the 1950s, there were not many reports on this topic, and it exponentially grew due to its spreading worldwide. In 2013 alone, there were 282 scientific publications listed in PubMed. After 2000, as the cases increased in India and other countries, the reporting and research focused on JE infection extensively. Further, as shown in Figure 1, we plotted data for the yearly publication count to understand the dynamics better.

## Materials and Methods:

## Genome Retrieval and Mining:

To obtain all the sequences of JE, we used the NCBI nucleotide database with the entry keyword -*Japanese encephalitis*' and obtained a total of 6945 sequence data files. All the data were downloaded and mined for the complete genome, and the rest were deleted. Further, we decided to consider the geographical locations, reporting year, and host organism for this study. After extensive manual mining, we found 29 genomic sequences of JE infecting different animals and reported from different geographical locations and used them for all the performed studies. Because of the difference in the reporting year and geographical locations, the JE virus from humans has taken seven times to cumulate the polymorphism among the various strains. After identification, we prepared a list of 29 genomics sequences belonging to the JE virus (Table 1). We renamed the serial number Accession Number sequence reporting country year to avoid any confusion during analysis and so on for the reader.

## Data analysis:

The genomes of the different species were downloaded through the NCBI plugin in Geneious Prime v.2019.2 (https://www.geneious.com) and reannotated through the local database's features, creating many new annotations [14]. Out of 29 sequences, the sequence reported from Malaysia with accession number HM596272.1 was the longest. We believed it would align all the sequences properly with the highest coverage; thus, it was taken as the reference sequence. The sequences were aligned using MAFFT aligner v.7.450 (https://mafft.cbrc.jp/alignment/server/) with the setting Auto algorithm selection and 200PAM/k = 2, Gap open penalty of 1.53, and all the data to find the distance between all the species [15- 16,17]. Annotation extractions provided a specific sequence that was further analysed and plotted. Transcription factors were predicted using the TRANSFAC (https://genexplain.com/transfac/) scan tool of EMBOSS (https://www.ebi.ac.uk/Tools/emboss/) [18, 19]. We have used the Phobos v.3.3.12 (https://www.ruhr-uni-bochum.de/spezzoo/cm/cm_phobos.htm) for the short sequence repeat analysis with the settings such as extending, where the exact search and the repeat unit length were kept to 1 bp (min) to 10 bp long, and the percentage of perfection was 0 to max [20- 21]. The extended sequence repeats (LSR) were analysed and extracted using the default program of repeat finder in Geneious Prime with the minimum repeat length of 100 and ignoring up to 10 bp and 0% mismatch, including different repeat sequences that were further categorised.

Again, all data were aligned using the MAFFT aligner v.7.450 to extract the single nucleotide polymorphisms (SNPs) separately in another column. Afterwards, we calculated the mutation percentage with the genome size for easy understanding [15- 17]. Mature peptides were predicted using Geneious Prime and reannotated with the local database [14]. Further, extracted data were plotted according to size and species names parallel to peptides. Signal peptides predicted using the Signal P-5.0 Server from the DTU Health Tech website (http://www.cbs.dtu.dk/services/SignalP/) [22]. The untranslated regions (UTR) were identified using the UTR scan, a server of ITB tools (http://itbtools.ba.itb.cnr.it/). Restriction sites were identified using the rebase database (http://rebase.neb.com/rebase/rebase.html) [23]. Further, all the annotation data were imported to Geneious Prime, and mutual annotations were performed from the local database that provides a vast list with the updated annotations, which were further exported for analysis in Excel and other tools. The phylogenetic tree was constructed using three programs and in three steps. Initially, we aligned the data using the MAFFT aligner because of its high speed and accuracy. We then plotted the tree using Geneious tree builder using the neighbour-joining method with the settings such as no defined outgroup and Tamura-Nei genetic distance model as parameters in the Geneious tree builder [14- 17, 24]. Further, the Itol server v.6 was used to modify the tree [25].

## Results:

## The Genome Length of *Japanese encephalitis*:

**Figure 2:**
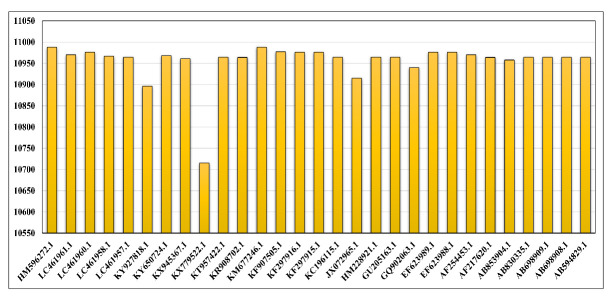
Comparative graphical representation for 29 sequences collected from NCBI, filtered based o different geographical locations, year of reporting, and most importantly, from the different hosts for comparative analytics and plotted with referencing to Accession number and sequence length.

The term -genome size II was erroneously attributed in 1976 by Ralph Hine Gardner in his research, even in discussions dealing specifically with terminology in this study area [26-27]. Genome size is the aggregate sum of DNA inside one copy of a solitary complete genome. A life form‘s multi-faceted nature is not legitimately relative to its genome size; total DNA content is broadly variable between organisms. Some single-celled organisms have substantially more DNA than humans, and the reasons for that are still unclear [28- 29]. However, genome size matters in multicellular organisms and their protein-coding genes. We collected 29 JE genomes reported from different geographical locations and plotted them in Figure 2 for a comparative understanding of the sequence length. All the genomes and their length were noted manually in the CSV file. HM596272.1 reported from Malaysia had the most extended sequence length, the reason behind taking the reference sequence, while KX779522.1 reported from China (Sichuan in 2016) has a sequence length of 10,715 bp. We have also taken three genomes reported from different regions of India. EF623989.1 and EF623988.1 have a sequence length of 10,976 bp and are reported from Maharashtra and Uttar Pradesh states. JX072965.1, reported from West Bengal, has a sequence length of 10,915 bp. While aligning the sequence, it was found that there is not much length variation in the sequences having sequence lengths of more than 10 kbps.

## Genomic annotations counts:

**Figure 3:**
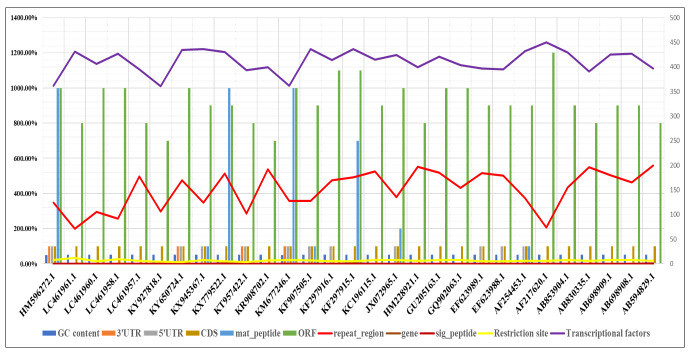
Graphical representation of count of whole-genome annotations, data includes the count of 3' UTR in the red-orange bar, 5' in the grey bar, CDS in the copper colour bar, a mature peptide in the ultramarine bar, ORF in the green bar, repeat region in the red colour line graph, a signal peptide in a carmine line graph, restriction site in a yellow line graph, transcriptional factors in the purple bar graph and % of GC in the blue colour bar.

The annotated genomes were kept in a separate folder, treated as a reference folder, and each sequence was reannotated while keeping it in the query folder of Geneious. This method was used to transfer the unique annotations of each sequence to another, resulting in more annotations to extend our study broadly. Further, the count of all annotations was extracted for comparative understanding. Among all 29 JE genomes, the GC content ranges from 50.1 to 52.1% of the total sequence length. KY927818.1 was reported in Cambodia in 2015 and had 52.1% GC content, the highest among all sequences. HM596272.1, KY650724.1, KT957422.1, and KM677246.1 are the four accessions that contain the untranslated regions at their 3' ends. HM596272.1, KY650724.1, KX945367.1, KT957422.1, KM677246.1, KF907505.1, KF297916.1, JX072965.1, EF623989.1, EF623988.1, AF254453.1 are the accessions that contain the untranslated regions at its 5′ ends (SS1). All 29 JE genomes have a single CDS. The count of mature peptides differed from 0-10 in all strains, and only HM596272.1, KX779522.1, and KM677246.1 had ten mature peptides in each strain. In Figure 3, genes, signal peptides, repeat regions, and restriction sites have been plotted for comprehension. We have also extracted transcription factor counts from all genome sequences.

## Analysis of transcription factors:

In molecular science, the succession explicit DNA-binding factor is called the transcription factor (TF), a protein that controls the pace of translation of genetic data from DNA to mRNA [30-31, 32]. TFs manage the genes by turning them on and off to ensure cell communication [33-34]. TFs work alone or with other protein complexes as activators or repressors to recruit RNA polymerase to specific genes [32,35]. We identified 1085 transcriptional factors in 29 genome sequences and classified them into forward and reverse directions. BAF1 and ECR have the highest length among all genomes. We added the TFs name, accession number, and length for comparative data visualisation. These transcriptional factors will help experimental laboratories working in gene-based drug design stop the mechanism of replication in viruses directly (SS2 and SF1).

## Comparative analysis of ORF from all JE genomes:

In molecular genetics, an open reading frame (ORF) is the piece of a perusing outline translated into protein. An ORF is a consistent stretch of codons that starts with a beginning codon (AUG) and closes at a stop codon UAA, UAG, or UGA [36-37,38]. We used Geneious Prime to identify ORF with the start codon TTG, CTG, ATG, and a standard genetic code. The Protein Coding Prediction graph can be combined with ORFs to identify coding sequences. We identified 313 ORFs from the 29 JE genomes (SS3). We then categorised the ORFs based on their direction in the sequence. Further, the data was plotted (SF2) for the concerned ac-cession number and name of the ORF. The length of the ORF, along with its direction, has been labelled on its bar.

## UTR regions of genomes:

**Figure 4:**
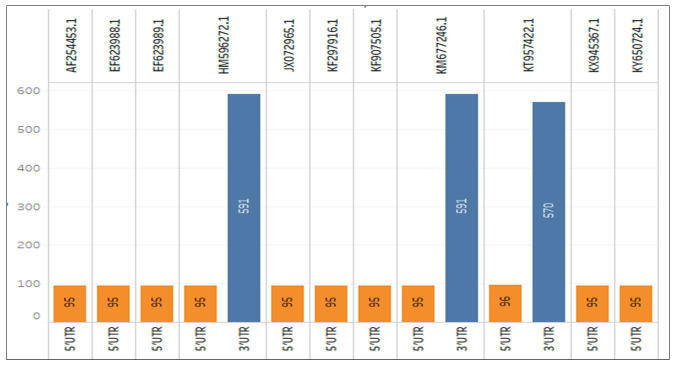
Untranslated Region of genomes is categorised and extracted based on their direction in the genome; 11 strains have 3'UTRs while only 3 strains show 5'UTRs. Data were plotted proportionately based on length and direction to the accession number and differentiated by colour.

In molecular biology, an untranslated region (UTRs) alludes to two segments; one on each side of the mRNA strand's coding sequence. When it is present on the 5′ sides, it is called the leader sequence; when it is present on the 3' sides, it is called the trailer sequence [39-40]. The mRNA is first transcribed from the compatible DNA arrangement and converted into a protein. A few locales of the mRNA are typically not converted into proteins in several cases. These locales are understood to be untranslated regions, e.g., 5' and 3' UTRs. We have identified the untranslated sequences and differentiated them into the genome's 3' and 5' UTRs. Further, we plotted the data in Figure 4 to compare the size and species containing the UTR and its type. We also depicted the length and respective name of the UTR with the accession and these UTRs from the above species helped categorise the genomic data into the fully functional and nonfunctional ranges to accelerate the translational analysis further. Only three genomes have 3' UTRs, and their accession numbers are HM596272.1, KM677246.1, and KT957422.1, and are of lengths 591, 591 bp and 570 bp, respectively. The rest of the genome has 5' UTRs with a length of 95 bp, except KT957422.1, with a length of 96 bp.

## Analysis of restriction sites on JE genomes:

The restriction sites on a DNA molecule contain explicit nucleotides, perceived by restriction enzymes, and are generally palindromic sequences. Restriction enzymes can cut the sequence between two nucleotides inside its acknowledgement site. The extracted data were categorised based on 3' overhang, 5' overhangs, blunt cutter, commercially available, commonly used, and plotted with different colours. All data was modified using colour and labelled with its length (SF3). Further complete data is available in SS4.

## Repeat analysis:

Repeat analysis is crucial due to its unique role in identifying genes or the location of biomarkers. Based on the length, we categorised the repeats into two essential types.

## Short sequence repeat analysis:

**Figure 5:**
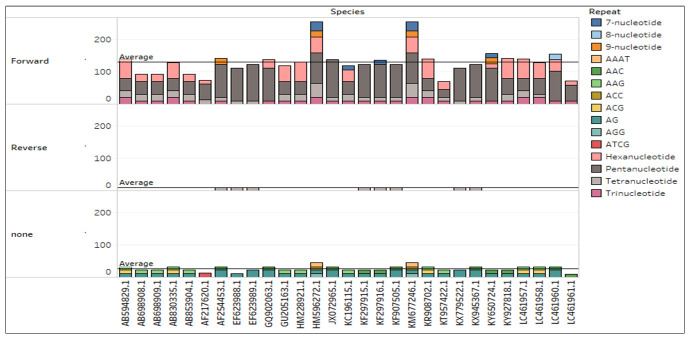
The extracted short sequence repeats from all *Japanese encephalitis* virus, plotted concerning SSR direction and differentiated with repeat colour. Each SSRs took as the sum of its repeat in particular JE strains.

In microorganisms, SSRs are elegantly linked to the modulation of gene expression, but in humans, unit number variability in SSRs is associated with specific genetic diseases [41-42]. Information on the functional limitations forced upon the SSRs reveals insights into their latent capacity using molecular clocks to check microbial genome advancement. Albeit microbial SSR genotypes are used with expanding recurrence to examine disease transmission and the development of microbial strains and secludes, such methodologies should be cautiously utilised. Microsatellites are DNA ex-tends of short, tandemly rehashed di-, tri-, tetra-or pentanucleotide themes [42]. Phobos led us to identify the tandem repeats in all genomes [20]. The longest repeat for the SSRs was set to 10 bp, and we extracted the repeats from every sequence. After extraction, we categorised them into forward and reverse. A few sequences were unidentifiable, so they have been put in the non-category. The 15 types of SSRs have been identified and plotted in Figure 5 with different colours, separated by the direction and accession number. Four SSRs fall under 9-nucleotide repeats, one in 8-nucleotide repeats, seven in 7-nucleotide repeats, sixty-four in hexanucleotide repeats, 173 in pentanucleotide repeats, 50 in tetranucleotide repeats, 32 in trinucleotide repeats (SS5).

## Long Sequence Repeats Analysis:

We identified long sequence repeats (LSRs) using the Geneious repeat finder and found LSRs in all 29 sequences. We extracted the data of LSRs and plotted them in tableau with the sum of LSRs concerning the species. The length of the longest LSR is 46,909 bp, identified in AB594829.1. Many LSRs were found within an LSR. Thus, its length is higher than its parent sequence length. The shortest is in the LC461961.1 with a sum of 123 bp. All the data is labelled on the bar graph in proportion to the accession number (SF4).

## Analysis of Peptides:

We Extracted and Categorised the Peptides into Signal and Matured Peptides

## Analysis of Signal Peptides:

A signal peptide or signal sequence is a short peptide at the N-terminal that incorporates residues inside specific organelles (ER, GA) discharged from the cell or embedded into most cell layers. Although most class I membrane-bound proteins have signal peptides, a large portion of type II and multi-spreading over membrane-bound proteins are focused on the secretory pathway by their first transmembrane space, which biochemically looks like a signal sequence, except it is not severed [43- 44]. Our study used Signal P-5.0 Server from the DTU Health Tech website to identify the signal peptides from all genomes [22]. We obtained signal peptides from only two sequences, AB594829.1 and AB830335.1, with same length of 276 bp.

## Analysis of Matured Peptides:

**Figure 6:**
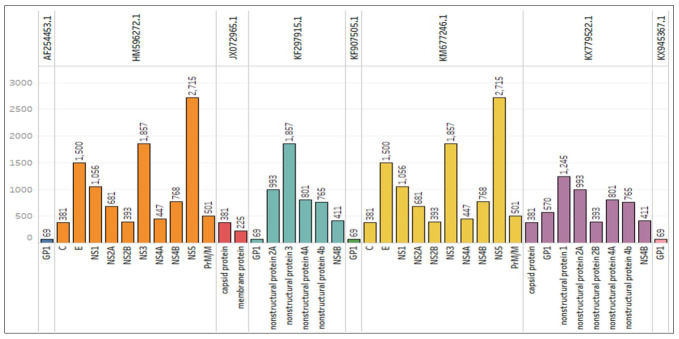
Extracted mature peptides were found in 8 strains plotted in different opacity ranges with an accession number-extracted data plotted with the sum of the length for each mature peptide broken down by accession numbers. Data have been labelled on each proportionally plotted mature peptide and accessions.

Mature peptides control infections, including their replication, transmission, pathogenicity, and host immunologic reactions [45-46]. We predicted mature peptide sequences from the entire genomes of 29 JE and obtained mature peptides from only eight sequences. The length of the mature peptides varies from species to species. KX945367.1, KX779522.1, KM677246.1, KF907505.1, KF297915.1, JX072965.1, HM596272.1, and AF254453.1 were found to contain mature peptides after prediction. We plotted the mature peptides extracted from the genomes. All accession numbers (mature peptides) have been plotted with a different colour. Data were taken as the name of the mature peptide and accession number and plotted proportionally in Figure 6. Further, the peptide length was taken and labelled on the bar. We found that the KX945367.1 and AF254453.1 have a single mature peptide with a 69 bp length. NS5 from HM596272.1 and KM677246.1 has the highest length of 2715 bp amongst all mature peptides. HM596272.1 was found to contain more mature peptides.

## Single Nucleotide Polymorphism:

**Figure 7:**
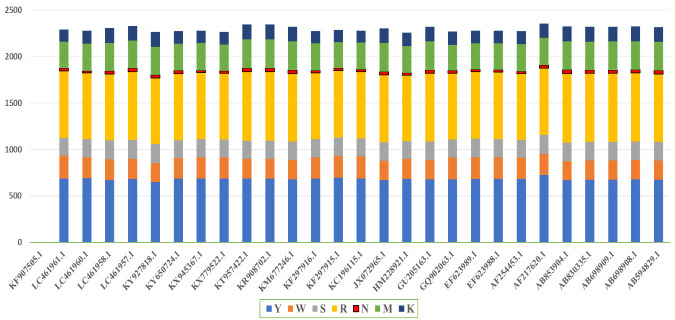
Single nucleotide polymorphism, HM596272.1, taken as reference. Y represents C is replaced by T (blue), M represents A replaced by C (green), W represents A replace by T (orange), K represents G replace by T (indigo), R represents A replace by G (yellow), S represents C replace by G (grey), N represents the deletion (red).

Single Nucleotide Polymorphisms (SNPs) are individuals' most widely recognised slight genetic variations. Every SNP addresses a distinction in a solitary DNA-building site called a nucleotide. An SNP may supplant the nucleotide cytosine (C) with the nucleotide thymine (T) in a specific stretch of DNA. SNPs are found regularly throughout an individual's DNA [47- 48]. They appear once in every 1000 nucleotides, implying about 4 to 5 million SNPs in an individual's genome [49-50,51]. These variations may be unique or happen in numerous people; researchers have discovered more than 100 million SNPs in populaces worldwide. SNPs can act as biological markers, helping researchers find disease-related genes [50]. When SNPs are located on genes or in a regulatory area near genetic material, they may play a significant role in infection by affecting the gene's function [41]. We have aligned the genomes individually with the reference species HM596272.1 and extracted the SNPs' count separately to calculate the percentage with the sequence length. We renamed the SNPs Y, which denotes T replaced by C (Table 2) for the graphical representation. Further, for conceptual understanding, we plotted the SNPs count from every species (SS6) data to understate its percentage and count for every SNP. We saw that R (denoting G replaces A) had the highest number of iterations in most genomes while M (A is replaced by C) had minor iterations. In Figure 7, we have highlighted the region to understand SNPs' count per species, particularly its type. KF907505.1 showed the least number of SNPs among all kinds, and this sequence only had 3Y (C is replaced by T), which is almost 0% (negligible) to the sequence length.

## Phylogenomics and divergence analysis:

**Figure 8:**
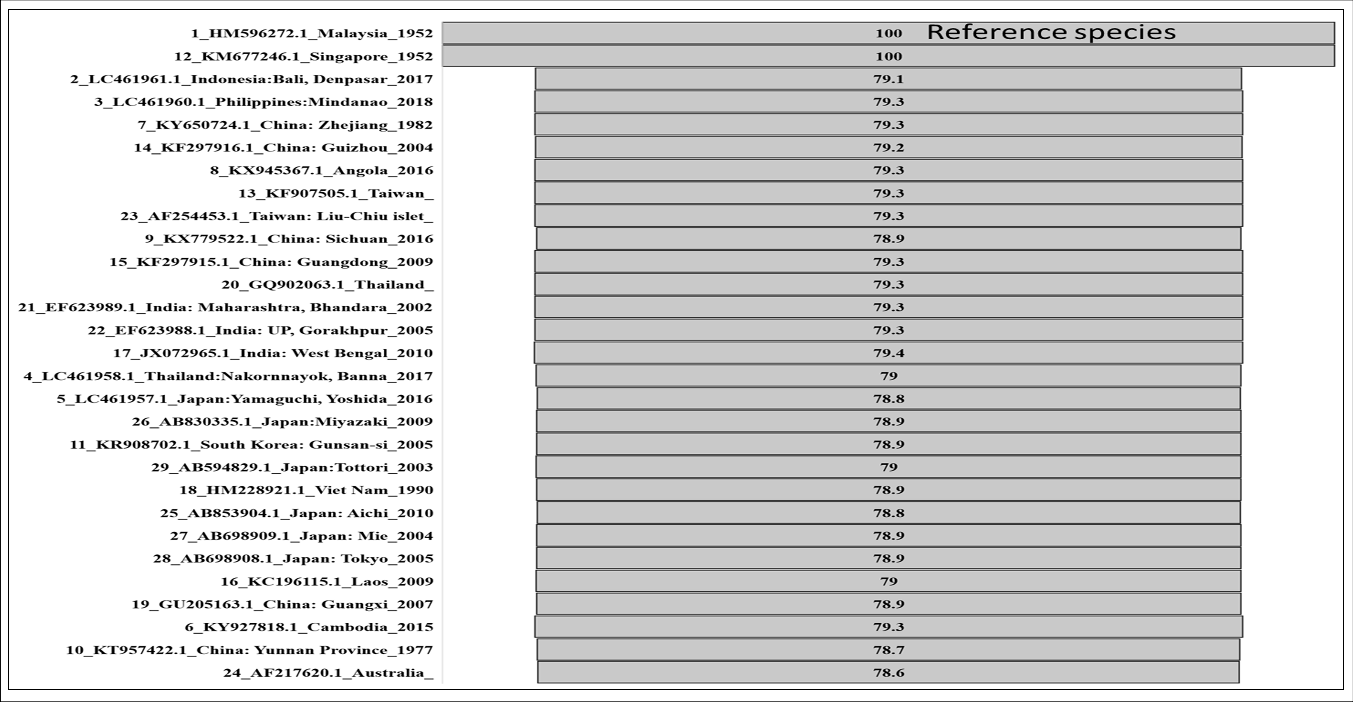
Comparative Distance plot among all organisms; Data were aligned using MAFFT aligner and calculated the distance in 2D matrices by taking HM596272.1 as a reference and calculating the exact amount of similarity with other species.

**Figure 9:**
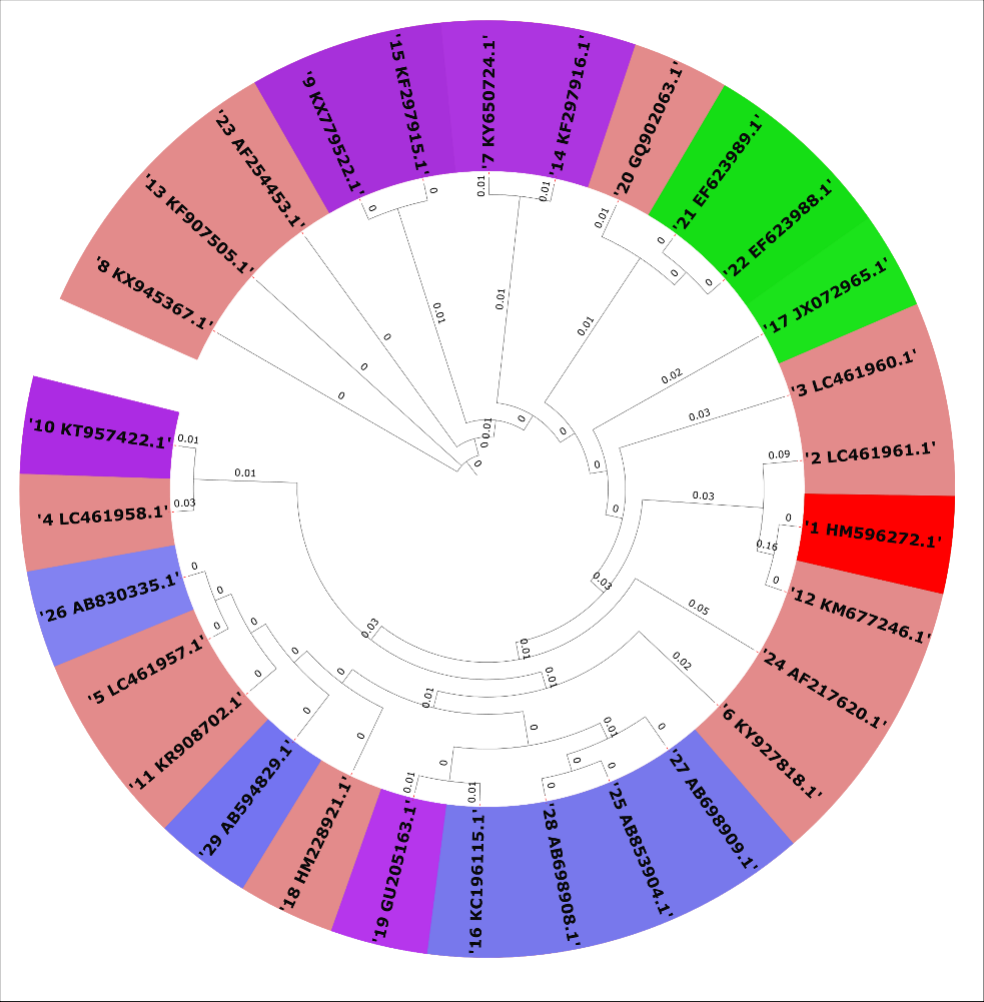
Phylogenetic trees for all *Japanese encephalitis* taken under study. The green colour shows species reported from India, the violet colour shows species reported from Japan, the sky-blue colour is those strains reported from China, and the red shows reference species reported from Malaysia. The further distance among all species is labelled for understanding the evolutionary aspects.

Genetic distance is the separation level (difference) between species or populaces estimated by some numerical strategy. Hence, the average number of codon or nucleotide variations per gene is a proportion of genetic distance [50, 52^53^]. When two species are distantly related, knowledge of the amino acid or nucleotide sequence is essential in phylogenomics. However, in the study of firmly related species or populaces, the impact of polymorphism cannot be ignored and must be studied in genes and proteins [53]. Hence, estimating the genetic distance between populaces regarding a function of allele frequencies for some genetic loci is standard. The calculated distance was added to a 2D matrix (SS7) and plotted in Figure 8 for comprehension. KM677246.1 has been reported from Singapore and has 100% similarity with the reference genome (HM596272). We plotted a funnel graph using the 2D matrix to quickly characterise the percentage of sequence similarity with the reference sequence. Most genomes showed 78-79% similarity among themselves. Strains reported from the Gorakhpur (UP) and Maharashtra states of India have a genome similarity of 79.3%, while the strain reported from West Bengal (India) had a similarity of 79.4%. We analysed all the various functionalities and structures of the genomes and aligned the data using MAFFT [15-17]. The Geneious tree builder file was exported further for final editing from the Itol server to plot it in a circular format to increase our understanding [25]. In the phylogenetic tree for all JE genomes taken under study, the green colour demonstrates species reported from India, the violet colour shows species reported from Japan, the sky-blue colour is for those from China, and the red colour shows the reference species reported from Malaysia (Figure 9). The distance among all species has been labelled along with the visuals to help understand evolutionary aspects.

## Discussion:

Genomes are sequences that have annotations on them that give information about sequences such as CDS, genes, transcriptional factors, GC contents, mature peptides, signal peptides, restriction sites, and untranslated regions, along with the size and position of the genomes [54]. These annotations are further used in the study of crop improvement or generation and in treating people with genetic disorders. While analysing the annotations, we took the GC content, untranslated regions (3' and 5'), CDS, mature peptides, ORF, repeat regions, genes, signal peptides, restriction sites on genomes, and transcriptional factors. The sequence reported from Cambodia (KY927818.1) in 2015 has the highest GC content, 52.1% of total genomic sequences. After comparing the complete genomes, we found only one CDS (with different lengths) in all sequences, while the count of mature peptides differs from species to species from 0-10 in each sequence. The transcription factors lead to replication and increased copy numbers of the virus in the human body; therefore, blocking TFs could also help tackle the epidemic. However, there are many complications and limitations associated with this method. Transcription factors contain at least one DNA-binding domain to attach to a specific DNA sequence adjacent to the genes regulating the functionality.

Out of 1085 identified TFs from 29 sequences, BAF1, ECR was found to have the highest length. We identified 313 ORFs in 29 JE genomes (SS3) and categorised them based on the direction in the sequence. A cumulative understanding of untranslated regions of the sequences and restriction sites gives an emerging idea to take genomes into the molecular biology lab to work from different angles and to test the effects of the drugs and UTR activity while treating with drugs. The short sequence repeats with less than ten bp length while the longer repeats were categorised under long sequence repeats are a better strategy to tackle the virus as the SSRs in microorganism plays crucial roles in gene activation, and the LSR can be used to detect the variants in molecular biology labs.

JE is not a new virus; massive sequences and protein structures are available in databases. Despite this, there is no direct cure for this virus. The strain identification can be made with single nucleotide polymorphisms. For this, we have extensively analysed and exported each SNP together for each sequence, and in future studies, it can be compared to the data of this report, which will be helpful for community-level detection and development in the JE genomes; this will also help scientists to characterise the level of divergence. While analysing the SNPs, we noticed that R (Table 2) had the highest number of iterations in most genomes, while M had minor iterations. In Figure 7, we have highlighted the region to understand the SNPs count per species, particularly its type. KF907505.1 showed the least number of SNPs among all kinds, and this sequence only had 3Y, which is almost 0% (negligible) to the sequence length.

## Conclusions:

This study aimed to take the genomes of JE from different geographical locations and hosts to infer the genomic similarity, divergence, and functional understanding of the genome. Our comprehensive, focused analysis of the genome annotations provided an intense and cumulative view at a single pace from a different angle, making it easier to identify the genome region, such as the transcriptional factors. Most importantly, the region of gene contents and the responsible ORF could also be identified. The aligned genome revealed that KF907505.1 (reported from Taiwan) only has 3Y (C is replaced by T) with the reference species, which means it does not have high mutations. Still, it vastly differs from other strains, and the same result shows almost 78-79.3% similarity with the rest of the genomes. We identified transcriptional factors with the drugs that could stop protein translation; ultimately, the T cell will identify that viral particle as a foreign pathogen and create a memory. Phylogenetic analysis also revealed that all JE are diverse, and the JE reported from India shares a very close relationship and is present in the same clade. The complete analysis, especially SNP and phylogenetic tree, suggests that JE reported from Taiwan and Malaysia has three almost negligible mutations. As most of the genomic content shared is by EF623989.1, other sequences reported from India seem to be the closest strains among all 29 considered genomic sequences. This comprehensive analysis will be helpful for researchers exploring genomic constraints. The data can be found in the supplementary sheets, starting with the proteomics level of mining and designing the target-based drugs against JE.

## Funding:

No Funding to report

## Institutional Review Board Statement:

N/A

## Data Availability Statement:

All the data and supplementary material can be published for future researchers.

## Author contributions:

ZMS, MAK, and MKW: Wrote the first draft; MYA, AMA, MAA, and AHA: literature survey; MMR: Data analysis and editing and reviewed the MS

## Figures and Tables

**Table 1:** Showing selected data‘s accession number, reporting country, and reporting year.

**S. No.**	**Accession**	**Reporting Country**	**Year**	**S. No.**	**Accession**	**Reporting Country**	**Year**
1	HM596272.1	Malaysia	1952	16	KC196115.1	Laos	2009
2	LC461961.1	Indonesia: Bali, Denpasar	2017	17	JX072965.1	India: West Bengal	2010
3	LC461960.1	Philippines: Mindanao	2018	18	HM228921.1	Viet Nam	1990
4	LC461958.1	Thailand: Nakornnayok, Banna	2017	19	GU205163.1	China: Guangxi	2007
5	LC461957.1	Japan: Yamaguchi, Yoshida	2016	20	GQ902063.1	Thailand	N/A
6	KY927818.1	Cambodia	2015	21	EF623989.1	India: Maharashtra, Bhandara	2002
7	KY650724.1	China: Zhejiang	1982	22	EF623988.1	India: UP, Gorakhpur	2005
8	KX945367.1	Angola	2016	23	AF254453.1	Taiwan: Liu-Chiu islet	N/A
9	KX779522.1	China: Sichuan	2016	24	AF217620.1	Australia	N/A
10	KT957422.1	China: Yunnan Province	1977	25	AB853904.1	Japan: Aichi	2010
11	KR908702.1	South Korea: Gunsansi	2005	26	AB830335.1	Japan: Miyazaki	2009
12	KM677246.1	Singapore	1952	27	AB698909.1	Japan: Mie	2004
13	KF907505.1	Taiwan	N/A	28	AB698908.1	Japan: Tokyo	2005
14	KF297916.1	China: Guizhou	2004	29	AB594829.1	Japan: Tottori	2003
15	KF297915.1	China: Guangdong	2009				

**Table 2:** Meaning of Single nucleotide polymorphism in Figure 7

**Type**	**HM596272.1**	**Query**
Y	C	T
M	A	C
W	A	T
K	G	T
R	A	G
S	C	G
N	-	-
